# Aryl Hydrocarbon Receptor (AhR) Modulates Cockroach Allergen-Induced Immune Responses through Active TGF**β**1 Release

**DOI:** 10.1155/2014/591479

**Published:** 2014-03-26

**Authors:** Yufeng Zhou, Sarah Mirza, Ting Xu, Priya Tripathi, Beverly Plunkett, Allen Myers, Peisong Gao

**Affiliations:** ^1^Johns Hopkins Asthma and Allergy Center, Johns Hopkins University School of Medicine, Baltimore, MD 21224, USA; ^2^Sher-i-Kashmir Institute of Medical Sciences, Medical College (SKIMS), Kashmir 190001, India

## Abstract

*Background*. Aryl hydrocarbon receptor (AhR), a multifunctional regulator that senses and responds to environmental stimuli, plays a role in normal cell development and immune regulation. Recent evidence supports a significant link between environmental exposure and AhR in the development of allergic diseases. We sought to investigate whether AhR plays a role in mediating cockroach allergen-induced allergic immune responses. *Methods*. AhR expression in human lung fibroblasts from asthmatic and healthy individuals and in cockroach extract (CRE) treated human lung fibroblasts (WI-38) was examined. The role of AhR in modulating CRE induced TGF**β**1 production was investigated by using AhR agonist, TCDD, antagonist CH122319, and knockdown of AhR. The role of latent TGF**β**1 binding protein-1 (LTBP1) in mediating TCDD induced active TGF**β**1 release was also examined. *Results*. AhR expression was higher in airway fibroblasts from asthmatic subjects as compared to healthy controls. AhR in fibroblasts was activated by TCDD with an increased expression of cyp1a1 and cyp1b1. Increased AhR expression was observed in CRE-treated fibroblasts. Importantly, CRE induced TGF**β**1 production in fibroblasts was significantly enhanced by TCDD but inhibited by CH122319. Reduced TGF**β**1 production was further confirmed in fibroblasts with AhR knockdown. Moreover, AhR knockdown inhibited CRE induced fibroblast differentiation. Furthermore, TCDD induced active TGF**β**1 release was significantly inhibited by LTBP1 knockdown. *Conclusion*. These results provide evidence for the role of AhR in modulating cockroach allergen-induced immune responses through controlling the active TGF**β**1 release, suggesting a possible synergistic effect between exposure to allergens and environmental chemicals on the development of allergic diseases.

## 1. Introduction

Asthma is the most prevalent serious chronic illness of children in the U.S [1]. While it is generally accepted that environmental chemicals and pollutants can contribute to the occurrence and exacerbation of asthma [2–5], the mechanistic links remain largely unknown. Specifically, environmental chemicals and pollutants have been shown to modulate environmental allergen-induced allergic diseases like asthma [6–8]. AhR is a multifunctional regulator that senses and responds to environmental stimuli and plays a role in normal cell development and immune regulation. It is known that dioxins and dioxin-like compounds, TCDD, PAH, and particulate matter (PM), can activate AhR, which then translocates to the nucleus and dimerizes with AhR nuclear translocator (ARNT). Within the nucleus, the AhR/ARNT heterodimer binds to xenobiotic responsive element (XRE) sequences and leads to changes in gene transcription (e.g., cyp1a1, cyp1b1) and a variety of toxicological effects, such as ROS generation, cell differentiation, and inflammatory cytokine production [9, 10]. Recent discoveries regarding AhR and environmental toxicant interaction and its influence on immune responses [11–14] highlight the potential link between environmental exposure and AhR in modulating allergen-induced allergic diseases. One intriguing and plausible hypothesis is that the expression of allergic and inflammatory disease could be attributable to those new immune “adjuvant” factors, for example, environmental chemicals, which often coexist with allergens and contribute to progressive fibrosis and pathological remodeling in asthma.

Indeed, recent studies have changed our understanding of asthma from that of a purely inflammatory disease to a disease in which both inflammatory and structural components are equally involved [15]. It has been suggested that there is a strong link between allergen-induced allergic airway inflammation and remodeling [16, 17]. In particular, exposure to cockroach allergen in early life can lead to allergic airway inflammation and an increased risk of developing asthma [18, 19]. One of the central components of airway remodeling is subepithelial fibrosis caused by deposition of collagen in asthma, a process in which fibroblasts and myofibroblast are critical. It has been shown that there is a correlation between the number of myofibroblasts and the degree of subepithelial fibrosis in the airway of asthmatic patients [20]. Of interest to us, allergens can induce an increase in the number of myofibroblasts [21], which may contribute to the progression of subepithelial fibrosis [22]. Recruitment of fibroblasts to the airway in asthma has been suggested to be potentiated by IL13 through a mechanism involving transforming growth factor-beta1 (TGF-*β*1) and MMPs [23].

TGF-*β*1 is produced by many cells within the lung, including fibroblasts, and plays a critical role in cell growth, differentiation, and immune regulation, and has been considered a principal mediator of airway remodeling [24–27]. Recent studies have demonstrated that disruption in TGF*β*1 signaling imposes a strong predisposition for human allergic diseases [28]. Specifically, increased active TGF*β*1 has been observed in airways from asthmatic patients [29] and from experimental mice during allergic airway inflammation [30] (Gao et al. JI 2014 in revision). Furthermore, increased TGF*β*1 activity appears to be controlled by latent TGF*β*-binding protein-1 (LTBP-1) [31]. Interestingly, primary mouse embryo fibroblasts from AhR−/− mice had increased expression of LTBP1 and higher levels of total and active TGF*β*1 that can be partially blocked by antibodies against LTBP1 [32], suggesting that AhR may control LTBP1 expression and subsequently activation of TGF*β*1 signaling. Although TGF*β*1 signaling and the AhR pathway have been well-studied, detailed information about the interaction between AhR and TGF*β*1 signaling remains largely unknown. Several mechanisms have been suggested regarding the regulation of AhR on TGF*β*1 signaling, including deregulation of TGF*β*1 secretion, suppression of TGF*β*1, or downregulation of the LTBP1 expression [11, 32, 33]. Furthermore, studies on ITE, an AhR agonist, have suggested that ITE can disrupt TGF*β*1 signaling by inhibiting the nuclear translocation of Smad2/3/4 and block TGF*β*1-induced myofibroblast differentiation and extracellular matrix production [34]. TGF*β*1, in turn, can suppress the AhR mediated gene expression through deregulation of AhR expression and/or localization [35]. Thus, studies on the cross-regulation between AhR and TGF*β*1 signaling might be essential for better understanding of the underlying mechanisms for the synergic effects between environmental chemicals and allergens on the development of allergic diseases. Specifically, we postulated that active TGF*β*1, released from fibroblasts or epithelium damaged by repeated environmental exposure, induces cell differentiation and immune regulation and is regulated by AhR.

In the present study, we have specifically focused on the functional significance of AhR in modulating cockroach allergen-induced immune responses through the release of active TGF*β*1. We found increased AhR expression in airways of asthmatic patients. Especially, AhR expression was increased in WI-38, a lung fibroblast cell line, after exposure to cockroach allergen. We then demonstrated that TCDD, an AhR agonist, can induce AhR specific downstream genes cyp1a1 and cyp1b1 expression. Importantly, we found the modulating effects of AhR on cockroach allergen-induced TGF*β*1 production and fibroblast differentiation. Finally, we demonstrated that TCDD-induced active TGF*β*1 was significantly blocked by LTBP knockdown. Taken together, we suggest that AhR plays a role in modulating cockroach allergen-induced immune responses by controlling the active TGF*β*1 release, and there is a possible synergistic effect between exposure to allergens and environmental chemicals on the development of allergic diseases.

## 2. Materials and Methods

### 2.1. Assessment of AhR in Human Airway

Paraffin-embedded human airway sections (5 *μ*m) from asthmatic (*n* = 4) and healthy individuals (*n* = 4) were used for immunofluorescence analysis. These human airway sections were provided by Dr. Allen (Johns Hopkins Asthma & Allergen Center). While it is not clear about the allergic status for those asthmatic subjects, all healthy individuals were nonallergic individuals. The human airway sections from three nonallergic heavy smokers also included controlling for AhR expression. In brief, nonspecific binding was blocked using 10% blocking serum in PBS for 1 hour. Sections were subsequently incubated with anti-human polyclonal antibody against Aryl hydrocarbon receptor (ab84833, 1 : 20, Abcam) and fibroblast marker antibody ER-TR7 (sc-73355, 1 : 50, Santa Cruz Biotechnology) overnight at 4°C. Normal rabbit IgG (Sigma-Aldrich) and rat IgG were used as a negative control. Sections were then incubated with Alexa Fluor 594-labeled goat anti-rabbit and FITC-labeled rabbit anti-rat secondary antibodies IgG at 1/100 dilutor for 2 hrs at RT. Nuclei were counterstained with 4′,6-diamidino-2-phenylin-dole, dihydrochloride (DAPI) (Sigma-Aldrich). Sections were subsequently dehydrated, mounted, and observed under the fluorescent microscope. The slides were evaluated using micrographs taken by a fluorescent microscope (Olympus BX-5). Imaging software (iVision; Biovision) was used to analyze areas of positive staining.

### 2.2. Flow Cytometry

WI-38 cells were fixed with BD Cytofix/Cytoperm solution for 30 min and then incubated with specific first antibody or isotype control for 30 min at 4°C in the dark. Then the cells were washed and incubated with fluorescent-conjugated second antibody. The following antibodies were used: anti-AhR (ab2770, Abcam) and anti-pSmad2/3(cell signaling). The samples were then analyzed on a FACSCalibur flow cytometer (BD Biosystems).

### 2.3. Immunocytochemical Analysis in WI 38 Cells

Cultured cells were fixed with 10% formalin at room temperature (RT) for 10 minutes and permeabilized for 5 mins with PBS containing Triton X100 and BSA buffer (0.3% TTX, 1% bovine serum albumin: TTX/BSA buffer). The cells were further blocked in 10% blocking serum for 30 min and then incubated with first antibody for 1 hour at RT. After washing with PBS, cells were incubated with fluorescent labelled secondary antibodies for 30 minutes at RT. Nuclei were counterstained with DAPI. Sections were subsequently dehydrated, mounted, and observed under the fluorescent microscope. The following antibodies were used: anti-AhR primary antibody (Abcam, ab2770, 1 : 20); anti-*α*-SMA (Abcam, ab32575); and anti-vimentin (eBioscience).

### 2.4. Quantitative Real-Time RT-PCR (qRT-PCR)

Total RNA from WI38 was extracted with RNAeasy kit (Qiagen, Valencia, CA). RT-PCR was performed using SYBR Green PCR Master Mix (Applied Biosystems, USA) according to the manufacturer's protocol. The primers for each gene were designed on the basis of the respective mRNA sequences so that the targets were 100–200 bp in length. Relative mRNA expression was calculated by normalization of all expression levels to actinand then compared to untreated control cells by the ΔΔCT method as described previously [36]. The following primers were used:

**Actin:** F, AGAAAATCTGGCACCACACC; R, CAGAGGCGTACAGGGATAGC;** AhR**: F, GTCGTCTAAGGTGTCTGCTGGA; R, CGCAAACAAAGCCAACTGAGGTG;** cyp1a1**: F, GATTGAGCACTGTCAGGAGAAGC; R, ATGAGGCTCCAGGAGATAGCAG;** cyp1b1**: F, GCCACTATCACTGACATCTTCGG; R, CACGACCTGATCCAATTCTGCC;** ltbp1**: F, TGAATGCCAGCACCGTCATCTC; R, CTGGCAAACACTCTTGTCCTCC;** ltbp-2**: F, CTGCACAGATGACAACGAGTGTC; R, AGAGTGTAGCCAGGGTAGCAGA;** ltbp-3**: F, CGGTCACTACAAGTGCAACTGC; R, CTTGTTCTCGCATTTGCCATCCG;** ltbp-4**: F, TTCCAGTGCAGGACCTGTCCTT; R: GAAGGAGCCTTCGGTGTTAGTG.

### 2.5. ELISA

Supernatants from cultured fibroblasts (WI38) were collected and measured for active TGF*β*1 by ELISA (eBiosciences), according to the manufacturer's instructions. Results were read with a Bio-Rad Bio-Plex instrument (Bio-Rad Laboratories, Hercules, CA).

### 2.6. Gene Knockdown by siRNA

Transcriptional knockdown was performed by transfection with siRNA oligonucleotide duplexes as a final concentration of 20 nM in DMEM using DharmaFECT transfection reagent (Thermo Scientific, Waltham, MA). A siRNA/transfection reagent complex was formed when siRNA and transfection reagent were mixed for 20 minutes. Then the transfection complex was added to each experimental well and incubated in the serum free media for 6 hours at 37°C. The transfection medium was replaced with complete medium and incubated at 37°C for an additional 24–48 hours. The gene knockdown was confirmed by qRT-PCR and western blotting. AhR specific siRNA was purchased from Thermo Scientific and LTBP1 specific siRNA was purchased from Sigma.

### 2.7. Western Blotting

Cells were washed twice with ice cold PBS and lysed in RIPA buffer containing protease and phosphatase inhibitor cocktails (Sigma). Protein content was measured with BCA reagent (Pierce). Equivalent protein samples were subjected to SDS-PAGE electrophoresis and then transferred to a polyvinylidene difluoride membrane (Millipore). After blocking with 5% nonfat dry milk in TBST, the membrane was incubated with primary anti-AhR (Abcam), anti-LTBP1 (GeneTex), anti-*α*-SMA (Abcam, ab32575), anti-vimentin (eBioscience), or anti-*β*-actin (clone C4, Santa Cruz) antibody as a loading control for normalization. Proteins reactive with primary Abs were visualized with HRP-conjugated secondary Ab and ECL reagents (Amersham). The levels of proteins were quantified by ImageJ (National Institutes of Health, USA) for the densitometric analysis of the band intensities and normalized to those of *β*-actin.

### 2.8. Statistical Analysis

Data are expressed as the means ± SEM for each group. Statistical significance for normally distributed samples was assessed using an independent two-tailed Student's* t*-test or with analysis of variance by using GraphPad Prism version 5.1 software (GraphPad Software, La Jolla, CA). Differences with *P* < 0.05 were considered statistically significant.

## 3. Results

### 3.1. Increased AhR Expression in Fibroblasts from Asthmatic Patients

**Figure 1:**
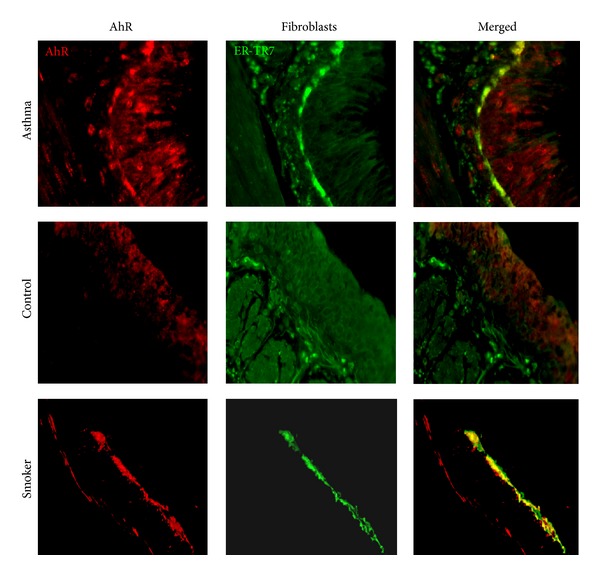
AhR expression in human airway. Immunofluorescence analysis of AhR expression in the airway, particularly fibroblasts from asthmatics (top), healthy individuals (middle), and heavy smokers (bottom), for antibodies against AhR (red) and fibroblasts marker (ER-TR-7, green). Figure 1 represents 4 individuals from each group.

To examine whether there was a differential expression for AhR in asthmatic and healthy individuals, we performed immunofluorescence analysis for both AhR and fibroblast marker ER-TR7 in human airway sections. Compared to healthy individuals (Figure 1, middle panel), the airway sections from asthmatic patients showed significant expression of AhR, increased fibroblasts marker ER-TR7, and thickening of basal membranes (Figure 1, top panel). Particularly, AhR was predominantly expressed in fibroblasts and basal membranes. Interestingly, significantly increased AhR expression was also observed in airway fibroblasts from heavy smokers (Figure 1, bottom panel). These findings suggest an increased AhR expression in fibroblasts from asthmatic patients and possibly from those who are repeatedly exposed to smoking.

### 3.2. Increased AhR Expression in CRE-Treated Human Lung Fibroblasts

**Figure 2:**
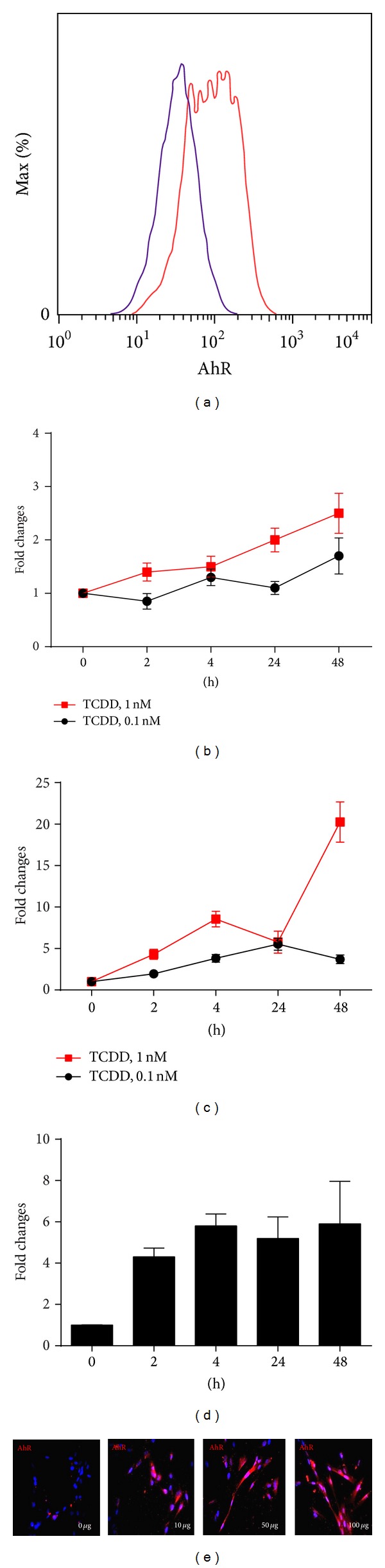
AhR expression in CRE-treated human lung fibroblasts. (a) AhR expression in WI-38 was detected using antibody against AhR (red line) and IgG2 (blue line) by flow cytometry. (b-c) Fibroblasts were treated with different doses of TCDD (0.1 nM and 1 nM) for 2 to 48 hours; expressions of cyp1a1 (b) and cyp1b1 (c) were examined by RT-PCR. (d) Fibroblasts were treated with 50 *μ*g/mL CRE for 2–48 hours and AhR expression was examined by RT-PCR. (e) Immunofluorescence analysis of AhR expression in CRE-treated fibroblasts at various doses (0–100 *μ*g/mL). Bars represent mean ± SEM of 3 independent experiments.

To delineate the role of AhR in the regulation of fibroblast's function and its mechanisms, we used human lung fibroblast cell line as an* in vitro* model. To validate AhR expression in fibroblasts, we detected AhR expression in WI-38, a human lung fibroblast cell line, by flow cytometry and western blot (data not shown). We found that AhR was constitutively expressed in fibroblasts (Figure 2(a)). We next examined whether AhR is functional; we treated fibroblasts using different doses of TCDD known AhR ligands (0.1 nM and 1 nM) for 2 to 48 hours; expression of AhR downstream genes cyp1a1 (Figure 2(b)) and cyp1b1 (Figure 2(c)) was examined by RT-PCR. Compared to those untreated fibroblasts, an increased expression was noted in TCDD treated fibroblasts for cyp1a1 in a dose- and time-dependent manner. There was nearly a 2-fold increase in cyp1a1 expression after treatment with 1.0 nM TCDD for 48 hours. Similarly, an 18.5-fold increase was observed for cyp1b1 when 1.0 nM TCDD was used to treat fibroblasts for 48 hours, suggesting that TCDD can activate the AhR pathway in fibroblasts. Furthermore, to investigate whether CRE can induce AhR expression, we treated fibroblasts with 50 *μ*g/mL CRE for 2–48 hours and AhR expression was examined by RT-PCR. An increased AhR expression was seen with a peak level at 4 hours for a 5.7-fold increase when compared with untreated cells (Figure 2(d)). Increased AhR expression in a dose-dependent manner was further observed by immunofluorescence analysis (Figure 2(e)), suggesting that cockroach allergen can induce AhR expression which may be critical in modulating allergen-induced immune responses.

### 3.3. AhR Modulates CRE Induced TGF*β*1 Production in Fibroblasts

**Figure 3:**
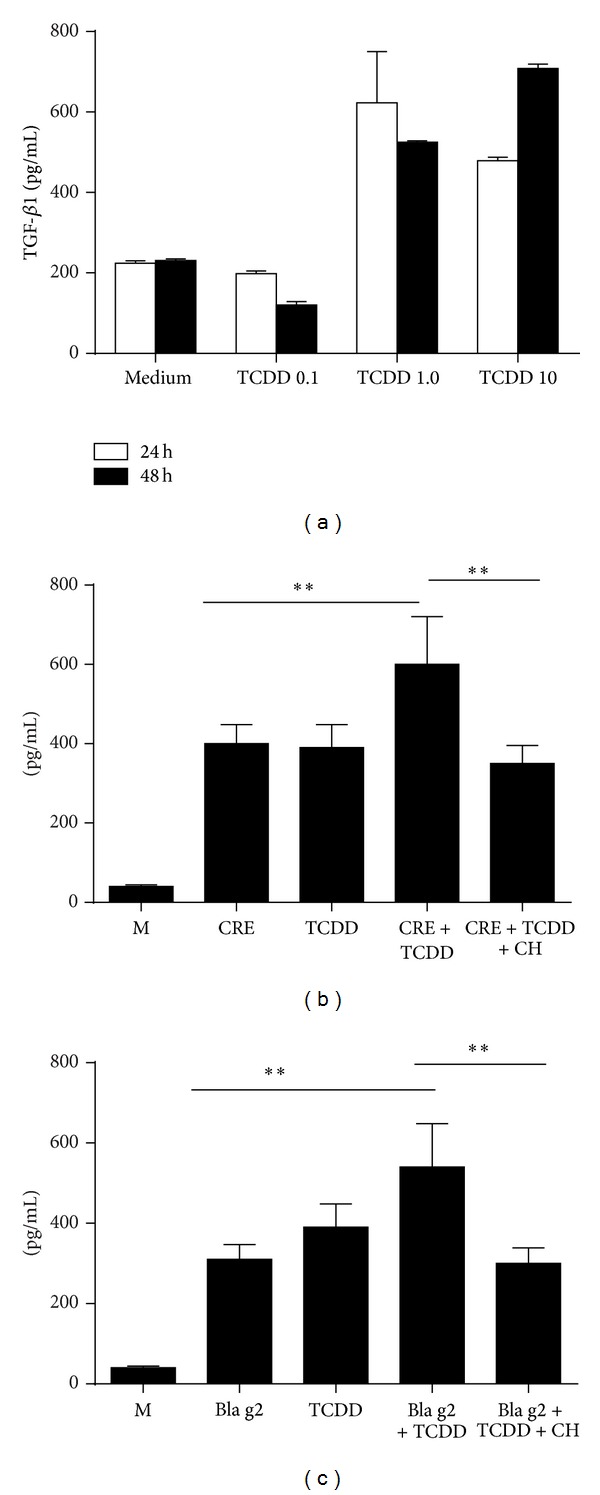
AhR modulates cockroach allergen-induced TGF*β*1 production. (a) TCDD can induce TGF*β*1 secretion by fibroblasts and (b) cockroach allergen-induced TGF*β*1 secretion by fibroblasts can be further enhanced by TCDD but inhibited by AhR antagonist CH122319 (c). (c) Cockroach allergen-induced TGF*β*1 production and the inhibitory role of CH122319 were further confirmed when Bla g2 was used. Bars represent mean ± SEM of 3 independent experiments. *P < 0.05, **P < 0.01.

To investigate whether AhR can modulate cockroach allergen-induced TGF*β*1 production that may control cell differentiation and immune regulation, we treated fibroblasts with CRE (50 *μ*g/mL) in the presence or absence of AhR agonist TCDD or antagonist CH122319 and detected the levels of active TGF*β*1 in supernatants of cultured and treated fibroblasts. We found that TCDD induced a significant release of active TGF*β*1 by fibroblasts that were treated with TCDD at various doses (1.0, 10 nM) and times (24, 48 hours, Figure 3(a)). Both cockroach allergen and TCDD as an individual induced increased levels of active TGF*β*1 by fibroblasts. Interestingly, the increased TGF*β*1 was further enhanced when both of them were combined (Figure 3(b)). In contrast, AhR antagonist CH122319 inhibited CRE+TCDD induced TGF*β*1 production (Figure 3(b)). Cockroach allergen-induced active TGF*β*1 release and the inhibition of CH122319 were further confirmed by using natural purified cockroach allergen, Bla g2 (Figure 3(c)). These findings suggest that AhR can modulate cockroach allergen-induced TGF*β*1 production.

### 3.4. Reduced Levels of Active TGF*β*1 in Fibroblasts with AhR Knockdown

**Figure 4:**
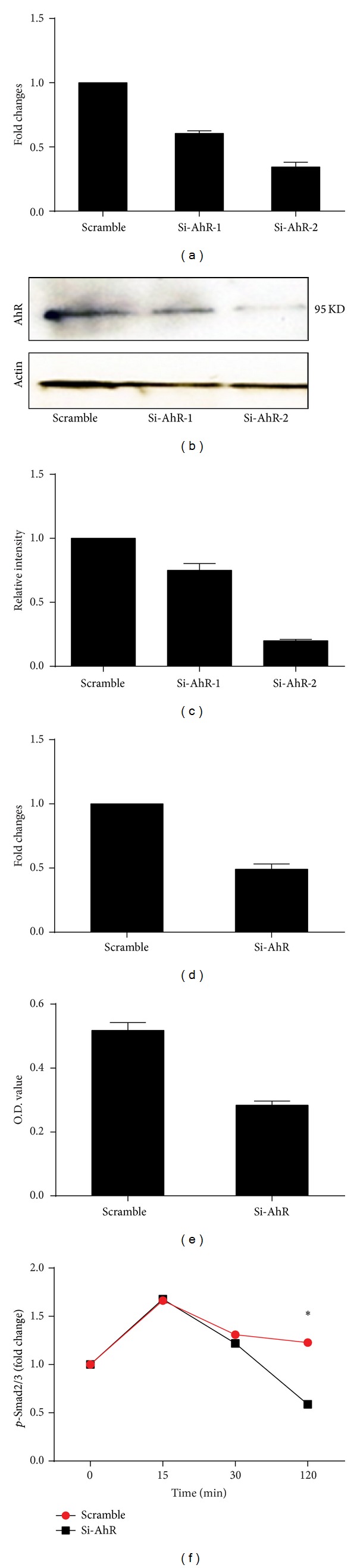
TGF*β*1 production was reduced in fibroblasts with AhR knockdown by siRNA. (a–c) AhR knockdown by siRNAs was confirmed by RT-PCR (a) and western blotting (b). (c) AhR expression in (b) was quantified by ImageJ and normalized to those of *β*-actin and then compared to scramble si-RNA treated group. (d) TGF*β*1 expression in fibroblasts treated with scrambled siRNA and si-AhR. (e) Levels of secreted TGF*β*1 stimulated with CRE by fibroblasts treated with scramble si-RNA and si-AhR. (f) WI38 cells were stimulated with 5 ng/mL TGF*β*1 for indicated time after AhR knockdown for 48 h; p-Smad2/3 was detected with flow cytometry. Bars represent mean ± SEM of 3 independent experiments. *P < 0.05, **P < 0.01.

To further examine the modulation of AhR on cockroach allergen-induced TGF*β*1 production, we knocked down AhR in fibroblasts using siRNAs. The AhR knockdown was validated by RT-PCR (Figure 4(a)), which showed at least 60% knockdown for si-RNA-2, and by western blotting (Figure 4(b)). AhR expression in Figure 4(b) was further quantified by ImageJ for the densitometric analysis of the band intensities and normalized to those of *β*-actin and then compared to scramble si-RNA treated group (Figure 4(c)). There was at least 70% knockdown for si-RNA-2. We next used fibroblasts with AhR knocked down by si-AhR-2 to detect TGF*β*1 expression at the RNA levels by RT-PCR (Figure 4(d)) and active TGF*β*1 secretion by ELISA (Figure 4(e)). We found that TGF*β*1 expression at the RNA level or in secretion was significantly reduced for fibroblasts with AhR knockdown. To examine whether AhR knockdown can affect the activation of TGF*β* signaling, we treated those fibroblasts with or without AhR knockdown with 5 ng/mL TGF*β*1 and measured phosphorylated Smad2/3 (p-Smad2/3) at various times by flow cytometry (Figure 4(f)). We noted an increased activation of Smad2/3 at 15 min for all these treated cells but we noted a decline at 30 mins and 120 mins. Interestingly, fibroblasts with AhR knockdown showed remarkable reduction in the levels of p-Smad2/3 at 120 mins as compared to the control cells. These data suggest that there may be a crosstalk between AhR pathway and TGF*β*1 pathway.

### 3.5. AhR Modulates CRE Induced Fibroblast Differentiation

**Figure 5:**
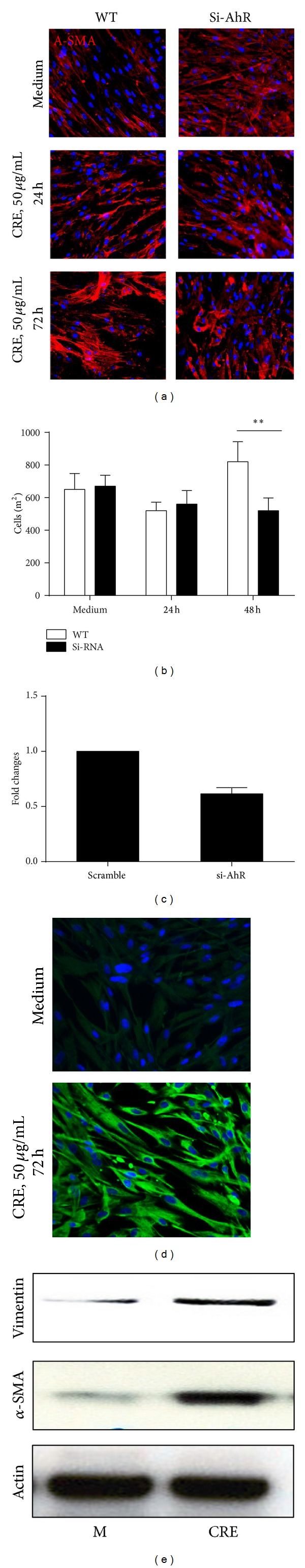
AhR controls fibroblast differentiation induced by CRE. (a) Differentiation of fibroblasts with and without AhR knockdown was evaluated by the expression of *α*-SMA with DAPI for nuclei immune-staining after cells were treated with CRE (50 *μ*g/mL) for 24 and 72 hours. (b) Positive staining for *α*-SMA was analyzed by Imaging software (iVision; Biovision). (c) *α*-SMA expression was detected by RT-PCR in fibroblasts with or without AhR knockdown. Bars represent mean ± SEM of 3 independent experiments, **P < 0.01. (d) Vimentin was detected by immune-staining after cells were treated with CRE (50 *μ*g/mL) for 72 hours. (e) Both *α*-SMA and vimentin expression were detected by western blots after WI38 cells were treated with 50 *μ*g/mL CRE for 72 h.

To examine whether AhR controls fibroblast differentiation induced by CRE, we cultured fibroblasts with and without AhR knockdown and treated with CRE (50 *μ*g/mL) for 24 and 72 hours. Differentiation of fibroblasts was evaluated by the expression of *α*-SMA with DAPI for nuclei immune-staining. While no clear change was noted in the *α*-SMA expression for fibroblasts with and without AhR knockdown at basal levels and 24 hours, an increased expression was seen when treated with CRE for 72 hours for fibroblasts without AhR knockdown. The *α*-SMA positive staining in fibroblasts was quantified and analyzed by Imaging software (Figure 5(b)). A significantly greater expression of *α*-SMA was observed for fibroblasts with CRE treatment. Finally, reduced *α*-SMA expression was detected by RT-PCR in fibroblasts with AhR knockdown as compared to those without gene knockdown (Figure 5(c)), suggesting that AhR may control cockroach allergen-induced differentiation. CRE induced fibroblast differentiation was further confirmed by immune-staining (Figure 5(d)) and western blots (Figure 5(e)) with another myofibroblast marker, vimentin.

### 3.6. Increased LTBP1 Expression in TCDD Treated Fibroblasts

**Figure 6:**
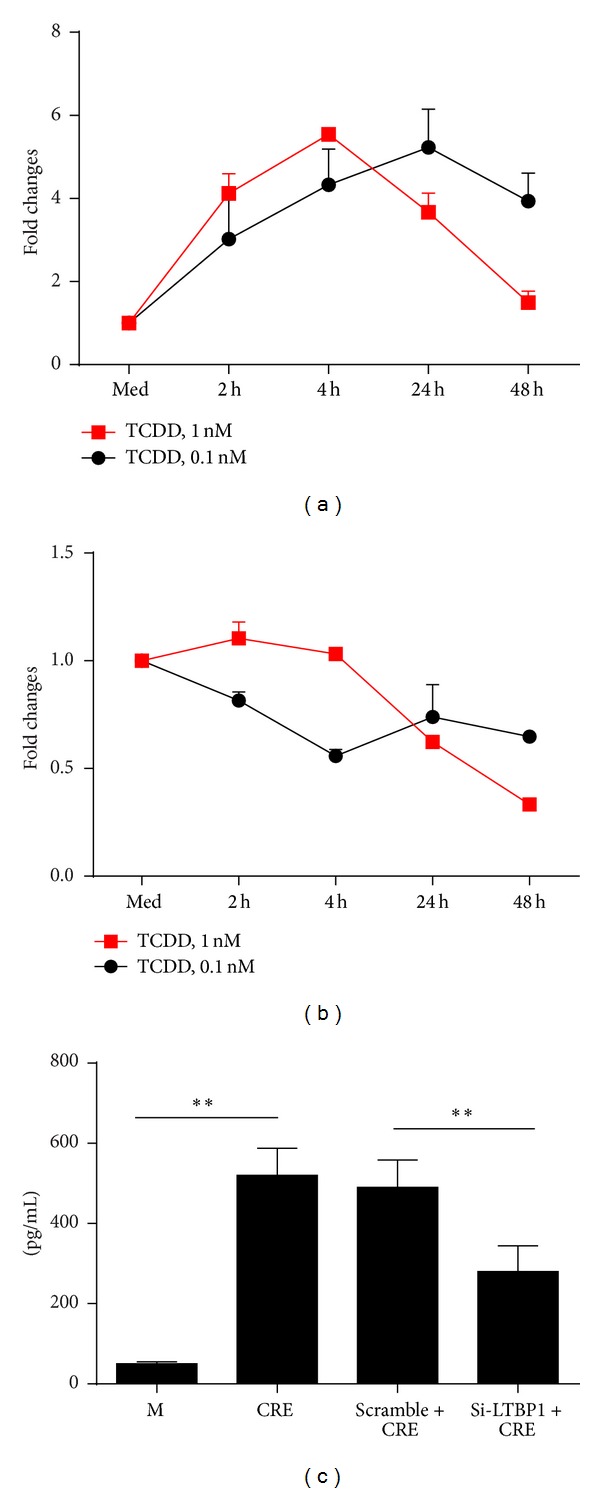
AhR controls LTBP-1 expression in fibroblasts. (a-b) Fibroblasts were treated with TCDD at 0.1 nM and 1 nM for 2 to 48 hours; expression of LTBP1 (a) and LTBP-2 (b) was detected by RT-PCR. Each point represents mean ± SEM of at least 3 independent experiments. (c) WI38 cells were treated with LTBP1 siRNA, scramble RNA, or medium control for 24 h and then treated with 50 *μ*g/mL CRE for 24 h; active form TGF*β*1 in the supernatant was detect with ELISA.

TGF*β*1 activity has been shown to be controlled by LTBP-1 [31]. Furthermore, it has been suggested that AhR may control LTBP1 expression, and subsequently activation of TGF*β*1 signaling [32]. To investigate whether the AhR ligand can activate LTBP1 that may control TGF*β*1 release, we treated fibroblasts using different doses of TCDD (0.1 nM and 10 nM) for different times (2 to 48 hours), and expression of LTBP1 to 4 at the RNA levels was measured by RT-PCR. Of these, increased LTBP1 was observed after treatment with TCDD (Figure 6(a)). The peak level was at 4 h for 0.1 nM and 24 h for 1 nM TCDD, respectively. Reduced expression of LTBP-2 was detected (Figure 6(b)), while no significant changes of LTBP-3 and 4 were detected (data not shown). To see the importance of increased LTBP-1 in AhR modulating active TGF*β*1 release, we pretreated fibroblasts with LTBP-1 specific siRNA. We found that TCDD induced active TGF*β*1 was significantly inhibited when LTBP-1 was knockdown (Figure 6(c)), suggesting that LTBP-1 may be critical in AhR controlling the release of active TGF*β*1.

## 4. Discussion

In the present study, we investigated the functional significance of AhR in modulating cockroach allergen-induced immune responses by controlling the release of active TGF*β*1. We found an increased AhR expression in the airways of asthmatic patients, mainly in airway epithelium and fibroblasts. While it is recognized that dioxins and dioxin-like compounds, TCDD, PAH, and PM, can activate AhR and lead to ROS generation, cell differentiation, and inflammatory cytokine production, we, for the first time, found that AhR ligands could enhance cockroach allergen-induced active TGF*β*1 production. The findings may suggest a possible biological link between environmental exposure and AhR in modulating allergen-induced allergic diseases. Because of the increased expression of AhR in epithelium, and predominantly in fibroblasts of the thickening basal membrane from asthmatic patients, it is possible that AhR as a sensor for environmental chemicals and allergens contributes to progressive fibrosis and pathological remodeling in asthma.

TGF*β*1 has been shown to be critical in cell growth, differentiation, and immune regulation, and a principal mediator of airway remodeling [24–27]. Our recent studies have observed an increased active TGF*β*1 in airways from cockroach allergen-induced mouse models (Gao et al., submitted 2013). In this study, we found that cockroach allergen can induce an increased production of active TGF*β*1 in fibroblasts. It is known that TGF*β*1 and AhR signaling pathways can crossregulate each other in a cell-specific manner [37]. We found that TCDD as an AhR agonist can enhance cockroach allergen-induced TGF*β*1 production, but CH122319 as an antagonist can inhibit allergen-induced TGF*β*1 secretion. TCDD that we used in this study has been considered as the most potent AhR ligand known and has been shown to be only slightly metabolized and to be relatively slowly excreted [38, 39]. The role of AhR in regulating cockroach allergen-induced TGF*β*1 release was further confirmed by using Bla g2, a purified cockroach allergen with undetectable endotoxin levels, and by using a human fibroblast cell line (WI38) with or without AhR knockdown. Particularly, the fibroblasts with AhR knockdown showed reduction of the levels of active TGF*β*1 as compared to the control cells. Furthermore, those cells with AhR knockdown showed remarkable reduction in the levels of p-Smad2/3. These findings suggest that AhR may be a positive regulator of cockroach allergen-induced TGF*β*1 signaling in human fibroblasts. Our findings seem to be contradictory to several previous studies suggesting a negative role of AhR in the initiation of food allergic responses [40, 41], regulation of TGF*β*1 secretion, and LTBP1 expression [11, 32, 42]. For instance, ITE, an AhR agonist, has been demonstrated to disrupt TGF*β*1 signaling by inhibiting the nuclear translocation of Smad2/3/4 and to block TGF*β*1-induced myofibroblast differentiation and extracellular matrix production [34]. In contrast, some other studies suggest that constitutive AhR activity positively controls TGF*β*1, TGF*β*2, and LTBP-1 in malignant glioma cells [43]. Thus, we postulate that there may be significant complexity of the regulatory mechanisms of AhR on TGF*β*1 signaling with coregulation of multiple other pathways.

A significant correlation has been observed between the number of myofibroblasts and the degree of subepithelial fibrosis in the airway of asthmatic patients [20], and fibroblasts have been recognized as major players by differentiation into myofibroblasts that may control the development subepithelial fibrosis in asthma. Further, TGF-*β*1 has been suggested to recruit fibroblasts to the airway in asthma [23]. We thus investigated whether AhR controls fibroblast differentiation after exposure to cockroach allergen. We found that, while there was no clear change in *α*-SMA expression (a marker for myofibroblasts) for fibroblasts with and without AhR knockdown at basal levels and at 24 hours, inhibited differentiation of fibroblasts was observed in AhR knocked-down fibroblasts with CRE treatment for 72 hours. This was further confirmed by showing a reduced *α*-SMA expression at the transcriptional level. Interestingly, a significant reduction in fibroblast proliferation was also noted at 72 hours (data not shown). Thus, we, for the first time, illustrate that AhR may control cockroach allergen-induced fibroblast differentiation that is critical in controlling subepithelial fibrosis and airway remodelling.

To explore the possible underlying mechanisms of AhR modulating allergen-induced TGF*β*1 signaling, we investigated LTBP1, which has been shown to be critical in controlling TGF*β*1 activity [31]. AhR has been shown to regulate Ltbp-1 transcription by a mechanism involving recruitment of coactivators such as CREB1 and corepressors such as HDAC2 to the Ltbp-1 promoter [44]. We thus hypothesized that AhR may regulate LTBP-1 transcription, control gene expression, and subsequently activate TGF*β*1 signaling [32]. Indeed, an increased LTBP1 was observed after treatment with TCDD with the peak levels of expression at early time points (4 h). In contrast, LTBP-2 expression was reduced, and LTBP-3 and 4 remained unchanged after treatment with TCDD (data not shown). Most importantly, AhR modulating active TGF*β*1 release was significantly blocked when LTBP-1 was knocked down. The findings support our initial hypothesis that LTBP-1 is critical in controlling AhR mediated TGF*β*1 secretion. Although the mechanism is not clear regarding the regulation of active TGF*β*1 by LTBP-1, studies have suggested that LTBP-1 contributes to TGF-beta1 activation, possibly through a process involving extracellular protease activities [31]. However, our findings in human fibroblasts showed that activation of AhR pathway can increase LTBP-1 expression, but in contrast, studies on the primary mouse embryo fibroblasts from AhR-/- mice also showed an increased expression of LTBP1 and higher levels of active TGF*β*1 [32]. So far, the reasons for the discrepancies between studies from human and mouse fibroblasts with or without AhR knockdown are largely unknown, a subject which would be of interest to pursue in the future.

**Figure 7:**
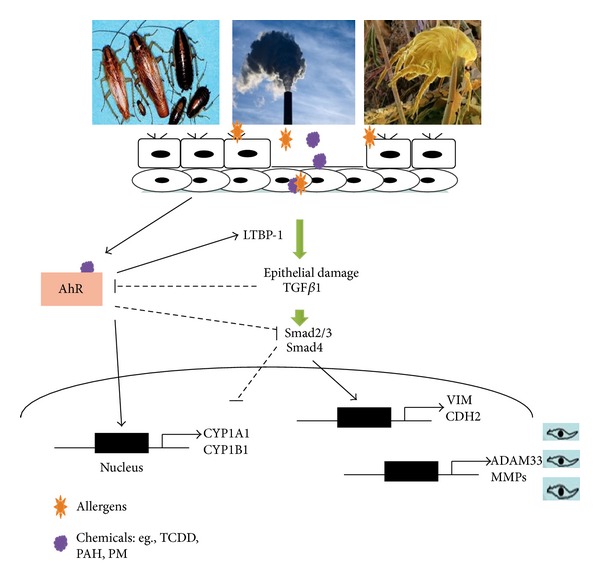
Proposed model of the role of AhR in modulating environmental chemicals and allergens induced activation of TGF*β*1 signaling (modified from an article by Denison and Nagy 2003) [38].

Taken together, this study provides evidence for the contribution of AhR to the mechanism regulating cockroach allergen-induced activation of TGF*β*1. In particular, studies on the interplay between AhR and TGF*β*1 pathways may better help us understand the potential mechanisms regarding the environmental chemical exposure modulating allergen-induced immune responses. As shown in Figure 7, we propose a model of the role of AhR in modulating environmental chemicals and allergen-induced activation of TGF*β*1 signaling (partially modified based on the article by Starsichova et al. 2012) [35]. Briefly, epithelial cells damaged by repeated exposure to environmental chemicals and allergens can release active TGF*β*1, which is largely controlled by LTBP-1. On the other hand, environmental chemicals bind AhR, leading to its activation, which plays a role in the control of LTBP-1 transcription, activation of TGF*β*1 signaling, and subsequently the control of cell proliferation, differentiation, and airway remodeling. These studies provide an important basis for a further detailed investigation of the interaction between AhR and TGF*β*1 signaling in environmental chemicals and allergens induced inflammation and repair/remodeling in asthma.

## Abbreviations

CRE: Cockroach extract
AhR: Aryl hydrocarbon receptor (AhR)
TCDD: 2,3,7,8-Tetrachlorodibenzo-p-dioxin
TGF*β*: Transforming growth factor *β*
cyp1a1: Cytochrome P450, family 1, subfamily B
cyp1b1: Cytochrome P450, family 1, subfamily B
LTBP-1: Latent TGF*β*-binding protein-1 (LTBP-1)
*α*-SMA: *α*-Smooth muscle actin.
